# Analysis of the composition and influencing factors of hospitalization expenses for 1517 patients with lung malignant tumors in Beijing

**DOI:** 10.1097/MD.0000000000037385

**Published:** 2024-03-15

**Authors:** Lili Zhao, Jiaji Tang

**Affiliations:** a Performance Office, Beijing TongRen Hospital, CMU, Beijing, China; b Medical Insurance Office, Beijing TongRen Hospital, CMU, Beijing, China.

**Keywords:** Beijing, hospitalization expenses, influencing factors, lung malignant tumor

## Abstract

This study aimed to analyze the composition of the total hospitalization expenses of patients with lung cancer in Beijing TongRen Hospital from January 2018 to December 2020 before and after the implementation of the “Beijing Medical Consumption Linkage Comprehensive Reform Implementation Plan” (hereinafter referred to as “Reform”). The SPSS 25.0 statistical software was used to perform descriptive statistics on the total hospitalization costs of selected 1517 patients with lung malignant tumors, and single factor and multivariate regression analysis were used to clarify the influencing factors of the patients’ total hospitalization costs. From 2018 to 2020, the total hospitalization costs of patients with lung malignant tumors increased year by year (*P* < .05), and the average length of hospital stay decreased year by year (*P* < .05). The total hospitalization expenses of patients with lung malignant tumors mainly include material expenses, surgical expenses, inspection expenses, inspection expenses and medicine expenses. After the implementation of the “Reform,” the proportion of medicine, inspection, nursing and other expenses in the total hospitalization expenses of patients with lung malignant tumors has been significantly reduced (*P* < .05), and the proportion of surgical expenses has been significantly increased (*P* < .05). The results of the univariate analysis showed that gender, age, length of stay in the hospital, surgery, and tumor location were the main factors affecting the total hospitalization expenses of patients (*P* < .05). The results of multivariate analysis showed that gender (female), age (<40 years old), length of stay (≥15 days), surgery (yes), and tumor location (right lung) are the main factors affecting the total hospitalization cost of patients with malignant tumors (*P* < .05). Under the premise of ensuring the efficacy of patients, the economic burden of patients is reduced by reducing the cost of materials, shortening the length of hospitalization, strengthening hospital management, and controlling the continuous growth of hospitalization costs.

## 1. Introduction

At present, China’s residents’ risk factors such as dyslipidemia rate, hypertension prevalence rate, obesity incidence rate, smoking rate, reduced physical activity, and other risk factors are maintained at a high level, and there is an upward trend, malignant tumors and other diseases caused by the loss of life of the residents may be further increased.^[1–3]^ In the case of China’s residents’ consumption capacity improvement, the security system is gradually improved, the demand for tumor prevention and treatment will still be gradually released, which will bring the continuous growth of tumor treatment costs. Relevant studies show that patients often need to receive radiotherapy or chemotherapy 6 to 7 times after resection surgery for malignant tumors, with a significantly higher average annual number of hospitalizations and higher per capita treatment costs than that of patients with all diseases.^[4]^ The accounting results show that the proportion of family health expenditure in oncology treatment costs is slightly less than 30% (the proportion of family health expenditure in outpatient oncology costs is close to 40%). Affected by the level of economic development, co-ordination efforts, financing methods, etc., the affordability of the health insurance fund in a certain region is relatively limited, from which the National Health Insurance Bureau has also clearly pointed out that: firstly, tumor-targeted drugs and other drugs need to be co-paid by the patients, and secondly, the health insurance fund has not been able to expand the scope of payment to cover non-therapeutic disease screening programs, including tumor screening and other non-therapeutic disease screening programs.

According to statistics, there were 2.37 million new cases of lung malignant tumors worldwide in 2021, which is the first among all cancer types, and its mortality rate is also about 22% of all cancer deaths.^[5]^ In some developed countries, lung malignant tumors are one of the most common cancers, accounting for the vast majority of national healthcare expenditures. The mortality rate of lung malignant tumors is also relatively high in our country compared to most countries.^[6]^ According to the data released by the National Cancer Center in 2019, it can be seen that in 2015, there were 787,000 new cases of pulmonary malignant tumors in China, with an incidence rate of 57.26/100,000, which is located in the 1st place of the incidence rate of malignant tumors; and the number of deaths from pulmonary malignant tumors in China in 2015 was 631,000, with a mortality rate of 45.87/100,000, which is located in the 1st place of the deaths from malignant tumors.^[7]^ It is expected that the mortality rate of lung malignant tumors in China will increase by 40% by 2030 compared with 2015, and the burden of disease is increasing.^[8]^ According to the statistical results of Cai et al^[9]^ it can be seen that in 2015, the hospitalization cost of China’s respiratory cancer patients was about 19.45 billion yuan RMB, and the expenditure of health insurance was 24.31 billion yuan RMB, accounting for 0.59% of the total health and health expenditures, and this figure continues to grow. On June 15, 2019, Beijing officially began to implement the Beijing Medical Consumption Linkage Comprehensive Reform Implementation Plan (hereinafter referred to as the “Reform”), which aims to reduce the price of test items; enhance the labor income of medical personnel; abolish the markup of disposable consumables; conduct bulk purchasing of medical consumables in conjunction with pharmaceuticals; and improve healthcare services and strengthen comprehensive supervision. It has been more than 2 years since the official implementation of this Reform policy, and this study focuses on analyzing the composition of hospitalization costs and influencing factors of 1517 patients with lung malignant tumors in a Beijing hospital from January 2018 to December 2020, to provide a basis for formulating interventions, alleviating patients’ economic burden, and better carrying out the guiding spirit of the Reform.

## 2. Materials and methods

### 2.1. Data sources and quality control

The data involved in this study came from the hospital discharge summary system of a hospital in Beijing. The main disease diagnosis (ICD-10: C34, malignant neoplasm of lung) on the discharge summary was used as the search criterion to retrieve all the information of hospitalized patients with malignant lung neoplasm from January 2018 to December 2020. Cases with hospitalization days longer than 90 days or hospitalization costs lower than 1000 yuan were excluded before analysis. Finally, 1517 patients with malignant lung neoplasm were included as the study subjects. The study was approved by the ethics committee of Beijing TongRen Hospital. Written consent was obtained from the participant’s parents or guardians.

### 2.2. Research methods

The medical records of hospitalized patients with malignant lung neoplasm from January 2018 to December 2020 in the hospital were collected, including the basic information of the patients (name, gender, age, etc.), the hospitalization-related information (admission time, hospitalization days, etc.), and the hospitalization cost information (drug cost, examination cost, laboratory test cost, treatment cost, surgery cost, etc.). The number of hospitalizations and related costs in each year were analyzed, and the composition of hospitalization costs in each year and the changes in the proportion of hospitalization costs before and after the implementation of the “Reform” were compared. The hospitalization costs were divided into drug cost, treatment cost, examination cost, laboratory test cost, surgery cost, material cost, nursing cost, and other costs. Univariate and multivariate analyses were performed to screen out the influencing factors of hospitalization costs for patients with malignant lung neoplasm.

### 2.3. Statistical analysis

SPSS 25.0 statistical software was used for statistical analysis. *T*-test was used for comparison between 2 groups, and one-way analysis of variance test was used for comparison among multiple groups. *T*-test or one-way analysis of variance test was used for univariate analysis, and multiple linear regression analysis was used for multivariate analysis. *P* < .05 indicated that the difference was statistically significant.

## 4. Results

### 4.1. General information of hospitalized patients with malignant lung neoplasm

A total of 1517 hospitalized patients with malignant lung neoplasm were included in this study, with an average age of 62.95 ± 3.47 years, including 829 males and 688 females, and an average hospitalization cost of 42,697.98 ± 3425.37 yuan (Table 1). From January 2018 to December 2020, the average hospitalization cost and daily hospitalization cost of hospitalized patients with malignant lung neoplasm increased year by year (*F* = 7.062, *P* < .05; *F* = 35.693, *P* < .05, Table 1), while the average hospitalization days decreased year by year (*F* = 15.340, *P* < .05).

**Table 1:** Statistics on the average length of stay and total cost of patients with lung malignant tumors from 2018 to 2020 (*n*, $\bar{x}$).

Year	Number of cases (M/F)	Average hospitalization days (d)	Average cost of hospitalization (yuan/)	Average daily hospitalization cost (yuan/)
2018	442 (252/190)	13.56	39,288.91	3070.49
2019	560 (297/263)	12.61	42,665.61	3503.14
2020	515 (280/235)	11.23	46,139.43	4248.96
Total	1517 (829/688)	12.42	42,697.98	3630.27

### 4.2. The composition of hospitalization costs for patients with malignant lung tumors

Among the hospitalization costs of hospitalized patients from January 2018 to December 2020, the proportions of drug costs, treatment costs, examination costs and nursing costs in the total costs decreased year by year, but the difference in the proportion of treatment costs in the hospitalization costs from January 2018 to December 2020 was not statistically significant (*F* = 0.043, *P* > .05), while the differences in the proportions of the other items were significant (*F* = 19.810, *P* < .05; *F* = 8.031, *P* < .05; *F* = 16.466, *P* < .05) (Table 2); the proportions of surgery costs and material costs in the hospitalization costs increased year by year, but only the difference in surgery costs was significant (*F* = 78.191, *P* < .05), and the difference in material costs was not statistically significant (*F* = 1.778, *P* > .05). The proportions of laboratory fees and other fees in the hospitalization costs increased in 2019 and were the lowest in 2020.

**Table 2:** Composition of hospitalization expenses for lung malignant patients from 2018 to 2020 ($\bar{x}$, *%*).

Category	2018		2019		2020	
	Cost (yuan)	Percentage (%)	Cost (yuan)	Percentage (%)	Cost (yuan)	Percentage (%)
Medicines	6815.63	17.35	5901.75	13.83	5457.08	11.83
Treatment	810.75	2.06	855.05	2.01	800.37	1.73
Inspection	6922.92	17.62	6308.75	14.79	6163.16	13.36
Laboratory	5455.87	13.89	6051.55	14.18	6173.67	13.38
Operation	1854.55	4.72	4469.19	10.47	5983.60	12.97
Material	15,435.24	39.29	16,921.93	39.66	19,703.74	42.70
Nursing	455.00	1.16	438.92	1.03	388.67	0.84
Other	1993.95	3.92	1718.46	4.03	1469.14	3.18
Total	39,288.91	100.00	42,665.61	100.00	46,139.43	100.00

### 4.3. The proportion of hospitalization costs for patients with malignant lung tumors before and after the implementation of the reform

According to the guiding spirit of the reform, the proportion of hospitalization costs for each category of patients with malignant lung tumors before and after the formal implementation of the reform policy in this hospital was analyzed. The results showed that after the implementation of the reform, the proportion of drug costs, examination costs, laboratory costs, material costs, nursing costs, and other costs in the hospitalization costs of patients with malignant lung tumors in this hospital decreased, among which the proportion of drug costs, examination costs, nursing costs, and other expenses decreased significantly (*t* = 6.340, *P* < .05; *t* = 3.337, *P* < .05; *t* = 4.329, *P* < .05; *t* = 2.754, *P* < .05), although the proportion of laboratory fees and material fees decreased, the difference was not significant (*t* = 1.880, *P* > .05; *t* = 0.329, *P* > .05) (Table 3). After the implementation of the reform, the proportion of treatment fees and surgery fees in the hospitalization costs of patients with malignant lung tumors in this hospital increased, among which the proportion of surgery fees increased significantly (*t* = 15.667, *P* < .05), and the increase in the proportion of treatment fees was not obvious (*t* = 1.445, *P* > .05).

**Table 3:** Composition of hospitalization expenses of patients with malignant lung before and after the implementation of the Reform ($\bar{x}$, %).

Category	Before the Reform (2018.1.1–2019.6.14)		After the Reform (2019.6.15–2020.12.31)	
	Cost (yuan)	Percentage (%)	Cost (yuan)	Percentage (%)
Medicines	6444.29	16.29	5670.90	12.45
Treatment	685.06	1.73	935.82	2.05
Inspection	6630.34	16.76	6282.65	13.80
Laboratory	5490.11	13.88	6267.32	13.76
Operation	1999.57	5.06	6021.84	13.22
Material	16,348.40	41.33	18,312.07	40.21
Nursing	436.53	1.10	418.45	0.92
Other	1521.48	3.85	1630.16	3.58
Total	39,555.79	100.00	45,539.21	100.00

### 4.4. Univariate analysis of the influencing factors of hospitalization costs for patients with malignant lung tumors

From the results of the univariate analysis, it can be seen that gender, age, length of hospital stay, whether surgery and tumor location significantly affect the hospitalization costs of patients with malignant lung tumors, and the difference is statistically significant (*P* < .05) (Table 4). This means that the mean hospitalization costs of these 5 variables at different levels have significant differences, rather than being caused by random errors. Specifically, in terms of gender, female patients have higher hospitalization costs than male patients; in terms of age, older patients have higher hospitalization costs than younger patients; in terms of length of hospital stay, patients with longer hospital stays have higher hospitalization costs than those with shorter stays; in terms of whether surgery, patients who receive surgical treatment have higher hospitalization costs than those who do not receive surgical treatment; in terms of tumor location, patients with unilateral tumor lesions have higher hospitalization costs than those with bilateral lesions. These results provide a basis for further exploring the influencing factors of hospitalization costs for patients with malignant lung tumors.

**Table 4:** Single factor analysis of influencing factors of hospitalization expenses for patients with lung malignant tumors from 2018 to 2020 (*n*, %, $\bar{x}$).

Influencing factors	Groups	Number of cases	Component ratio (%)	Average cost	*t/F*	*P*
Sex	Male	829	54.65	39,132.9	5.691	0
	Female	688	45.35	47,353.4		
Age (yr)	<40	41	2.71	46,078	1.561	.003
	40–59	477	31.44	45,346.8		
	60–79	921	60.71	42,826.8		
	≥80	78	5.14	26,373		
Days of hospitalization	<15	1078	71.06	37,676	15.685	0
	15–29	413	27.22	53,350.7		
	30–44	20	1.32	88,711.2		
	≥45	6	0.4	99,566.8		
Surgery or not	Yes	990	65.26	55,208.7	28.952	0
	No	527	34.74	19,665.3		
Tumor site	Right lung	725	47.79	45,963.5	59.179	0
	Left lung	535	35.27	46,749.3		
	Bilateral lungs	257	16.94	26,015		

### 4.5. Multifactorial analysis of factors influencing hospitalization costs for patients with lung malignancies

We further used univariate analysis and multivariate linear regression analysis to explore the main factors affecting the hospitalization cost of lung cancer patients. The results of univariate analysis show that gender, age, length of hospital stay, whether surgery, tumor location, tumor stage, metastasis, comorbidity, and complication have significant effects on the hospitalization cost of lung cancer patients (Table 5). To exclude the influence of other confounding factors, multivariate linear regression analysis was performed on the results of univariate analysis. The results of multivariate linear regression analysis after controlling variables show that gender (female), age (<40 years old), length of hospital stay (≥15 days), whether surgery yes and tumor location (right lung) are the main factors affecting the hospitalization cost of lung cancer patients (Table 6). These factors have different effects on hospitalization costs, among which length of hospital stay has the greatest impact, followed by surgery, then age, and finally gender and tumor location. The research results of this paper provide a valuable reference for the medical cost management of lung cancer patients.

**Table 5:** Multiple regression analysis assignment table.

Influencing factors	Variable code	Assignment	Meaning
Hospitalization costs	Y		
Sex	X1	A1	Male
		A2	Female
Age (yr)	X2	B1	<40
		B2	40–59
		B3	60–79
		B4	≥80
Days of hospitalization	X3	C1	<15
		C2	15–29
		C3	30–44
		C4	≥45
Surgery or not	X4	D1	Yes
		D2	No
Tumor site	X5	E1	Right lung
		E2	Left lung
		E3	Bilateral lungs

**Table 6:** Multivariate analysis of factors affecting hospitalization expenses of patients with lung malignant tumors from 2018 to 2020.

Variant		B	Standard error	*β*	*t*	*P*	95% confidence interval for B	
							Lower limit	Upper limit
Sex	Male	8220.49	1444.36	0.145	5.691	0	5389.61	11,051.4
	Female							
Age (yr)	<40							
	40–59	−731.15	4562.52	−0.012	−0.16	.873	−9680.7	8218.39
	60–79	−3251.12	4474.63	−0.056	−0.727	.468	−12,028	5526.01
	≥80	−19,704.9	5407.86	−0.154	−3.644	0	−30,313	−9097.2
Days of hospitalization	<15							
	15–29	15,674.67	1544.65	0.247	10.148	0	12,644.8	18,704.5
	30–44	51,035.17	6023.53	0.206	8.473	0	39,219.8	62,850.5
	≥45	61,890.71	10,927.1	0.137	5.664	0	40,456.9	83,324.5
Surgery or not	Yes	−35,426.8	1226.82	−0.60	−28.88	0	−37,833	−33,020
	No							
Tumor site	Right lung							
	Left lung	785.853	1554.12	0.013	0.506	.613	−2262.6	3834.31
	Bilateral lungs	−19,948.5	1979.55	−0.265	−10.077	0	−23,831	−16,066

## 5. Discussion

Since the 1970s, China’s incidence and mortality rates of malignant tumors have been on a clear rise, with lung cancer becoming the main cause of cancer deaths among Chinese residents, ranking first in both incidence and mortality.^[10]^ The number of hospitalized patients with lung malignancies in this hospital increased in 2019 compared to 2018, and decreased in 2020 compared to 2019, but overall still showed an increasing trend, consistent with the increasing trend of lung cancer incidence.

According to the current diagnosis and treatment guidelines for lung malignancies, most patients require radiotherapy or chemotherapy. From 2018 to 2020, the shortest hospital stay for patients with lung malignancies in this hospital was 1 day, the longest was 84 days, and the average length of stay was 12.42 days. Under the positive guidance of relevant policies, this hospital gradually standardized its diagnosis and treatment practices, and the average length of stay showed a downward trend over 3 years. Although reducing the length of stay can theoretically reduce hospital costs, the average hospital costs of patients with lung malignancies in this hospital increased year by year from 2018 to 2020, directly leading to an increase in daily hospital costs, which may be due to the application of new treatment methods in clinical practice. Reducing patient length of stay in situations where beds are tight is of great significance for improving hospital economic efficiency, reducing patient bed waiting time and reducing medical disputes, but relevant surveys show that some medical institutions pursue reducing length of stay, and there are phenomena such as urging patients to discharge or handling secondary admissions, which may affect the necessary medical needs of patients with serious conditions.

It is very important to master the composition of hospitalization costs paid by hospitalized patients in order to control the situation of excessive hospitalization costs. From the statistical results of this study, it can be seen that the proportion of drug costs in the hospitalization costs of patients with lung malignant tumors from 2018 to 2020 decreased year by year, and the proportion of treatment fees, examination fees, and nursing fees in the hospitalization costs of this hospital also decreased year by year; the proportion of surgery fees and material fees in the hospitalization costs increased year by year; the proportion of laboratory fees and other fees was the highest in 2019 and the lowest in 2020. The medical consumables linkage reform in Beijing pointed out that medical consumables and drugs should be jointly purchased in large quantities to effectively reduce the costs of consumables and drugs; cancel the markup of disposable consumables; reduce the cost of instrument examination; increase the price that reflects the labor value of medical staff; improve the quality of medical services and strengthen comprehensive supervision. This study analyzed the composition of hospitalization costs of patients with lung malignant tumors before and after the implementation of the “Reform.” Compared with before the implementation of the “Reform,” the proportion of treatment fees and surgery fees for hospitalized patients increased, but the increase trend of treatment fee proportion was not obvious; the proportion of drug fees, examination fees, laboratory fees, material fees, nursing fees, and other fees for hospitalized patients decreased, but the decrease of laboratory fee and material fee proportion was not obvious. The insignificant decrease in laboratory fee and material fee proportions may be due to the fact that after relevant policies and formal implementation of the “Reform,” relevant laboratory costs and consumable costs were reduced, making patients more inclined to use better materials and more examinations; in addition, doctors tend to choose better consumables within the economic affordability range of patients in order to ensure treatment results. The “Reform” proposed to increase the price that reflects the labor value of medical workers, but the proportion of nursing fees for patients with lung malignant tumors in this hospital decreased significantly; under normal circumstances, nursing fees and bed fees are calculated by day in the actual settlement process. The analysis results of this study show that there is a strong positive correlation between length of stay and nursing fee (*R* = 0.925, *P < *.05), that is, reducing the average length of stay will directly reduce nursing costs, which may be the main reason for reducing nursing fee proportion.

The composition of hospitalization costs paid by hospitalized patients directly reflects the current situation of cancer prevention and treatment in China, and there are the following problems: First, the lack of investment in prevention, the cost mainly occurs at the end of treatment, and the proportion of hospitalization costs is prominent. At present, the proportion of prevention costs in recurrent health expenditures in China is only about 7%. The proportion of prevention services in the prevention and treatment costs of diseases such as malignant tumors is relatively low, and a large amount of health resources are used for the treatment of middle and late-stage patients, with high consumption and low efficiency. Second, the population concentration of costs is significant. According to relevant research, the incidence rate of malignant tumors in the population aged 0 to 39 is at a low level, and it rises rapidly after 40 years old, reaching its peak at 80 years old.^[11]^ Correspondingly, China’s cancer treatment costs also show a significant concentration in the population aged 45 to 74. Third, the consumption of costs is mainly concentrated on cancer (83.44%), especially incurable cancer. Relevant statistics show that lung cancer, liver cancer, and stomach cancer rank first in the cause of death. These cancers have high mortality rates and bring large consumption of treatment costs.^[12,13]^ From the perspective of cost accounting results, China’s cancer treatment costs are mainly consumed by lung cancer, breast cancer, stomach cancer, liver cancer, etc., and the health output effect of resource input is poor.^[14]^ Fourth, the institutional flow direction of costs reflects that the role of grassroots in China’s cancer prevention and treatment system is insufficient. The responsibility orientation of primary medical and health institutions is to provide rehabilitation and nursing services for patients with clear diagnosis during stable period and recovery period. But the fact is that the role of primary medical and health institutions in cancer prevention and treatment management has not been fully played. Due to the high technical requirements for cancer prevention and treatment management, coupled with the lack of equipment and talents in primary medical and health institutions, patients with cancer have a low willingness to seek medical treatment at the grassroots level.^[15]^ Relevant research also points out that at present, rehabilitation after hospitalization for malignant tumors and screening before diagnosis are all concentrated in secondary and above medical institutions.

As can be seen from Tables 2 and 3, material costs, examination fees, laboratory fees, surgery fees and drug fees are the main components of hospitalization costs for hospitalized patients, among which material costs account for the highest proportion. To reduce material costs, medical staff must adhere to strict diagnosis and treatment guidelines, and choose appropriate examination items and consumables according to the actual situation of patients in their actual work, guiding the reduction of hospitalization costs for patients from the source of doctors; on the other hand, hospitals should strengthen the management of departments, refine to individuals, and include the proportion of material costs in the performance appraisal of medical staff within the department, and control the use of unnecessary medical consumables.

To control the continuous growth of medical costs, it is necessary to adjust the cost structure. At present, there are still phenomena such as “supporting medicine with medicine” and “supporting medicine with equipment” in some medical structures, resulting in a high proportion of laboratory fees, examination fees and drug fees. In contrast, the proportion of nursing fees and treatment fees in hospitalization costs is low. The low charging standard of treatment fees and nursing fees reflects the public welfare nature of China’s hospitals, but this nature cannot scientifically and reasonably include the technical labor costs of medical staff into medical costs, making the price inconsistent with the technical labor value. To solve the above problems, the government can cooperate with hospitals to carry out regular inspections of medical services and drug costs, and strengthen the supervision of the operation behavior of medical institutions; through evidence-based medicine methods, find alternative drugs with better effects but relatively low prices, standardize the dosage and course of medication; establish an effective compensation mechanism, appropriately increase the technical service charges of medical staff, so that their income can truly reflect their medical value.

The work of reducing average length of stay has been carried out for many years in China and has achieved good results. Average length of stay is an indicator that reflects hospital work efficiency. Under the premise of ensuring patient treatment effect, shortening patient’s average treatment days can achieve a win-win situation for society, hospital and the patient. The results of multifactor analysis show that the hospitalization cost of patients with lung malignant tumors with a length of stay ≥ 15 days is significantly higher than that of patients with a length of stay < 15 days, that is, length of stay is one of the main factors affecting hospitalization cost. In the actual work of shortening average length of stay, hospitals can reduce patient waiting time for examination by formulating corresponding policies; strengthen department management, evaluate daily medical work and various clinical nursing operations in each department, carry out health economics evaluation, and provide scientific basis for hospitals and relevant management departments to formulate corresponding policies.

Cancer can significantly reduce patient’s medical expenses if detected early and treated. Liu Zhaorui et al’s study showed that the cost of traditional therapy for esophageal cancer was 1.3 times that of family total income, while the cost of early diagnosis and early treatment for esophageal cancer was only 1/8 that of family total income. The benefit/cost ratio was 4.0, which had good economic and social benefits.^[16]^ If multiple departments jointly develop early diagnosis and early treatment methods for lung malignant tumors, combined with clinical diagnosis and treatment guidelines for lung malignant tumors, they can provide patients with optimal diagnosis and treatment plans. Under the premise of ensuring patient treatment effect, unnecessary examination and laboratory items can be reduced to reduce patient’s economic burden.

The results of multifactor analysis in this study show that female lung malignant tumor patients have significantly higher hospitalization costs than male patients. In the past few decades, many scholars have confirmed that smoking is a major risk factor for lung cancer incidence. With China’s social changes and changes in fashion trends, the number of female smokers has increased year by year. At the same time, due to the lack of social norm management, women have become the biggest victims of passive smoking. In addition, women are also harmed by indoor cooking fumes for many years, leading to a rapid increase in female lung malignant tumor incidence. As a professional institution providing professional medical services, hospitals should regularly carry out popular science education, propose effective measures from social and personal aspects to reduce female lung malignant tumor incidence and mortality rate.

There are several limitations should be noted. First, this is only a single-center study. The conclusions may be biased. Second, the data sources are limited. Some factors that may affect hospitalization costs such as family income, family members, marital status, ethnicity, etc. were not included in the analysis. Third, due to the particularity of the medical environment in Beijing, some patients who came to Beijing for medical treatment from other places could not enjoy medical insurance, so this study did not discuss the actual total cost after medical insurance reimbursement. Current conclusions could be verified and strengthened in other cities, and only consider the participants with or without medical insurance.

## 6. Conclusions

Our study demonstrates that, under the premise of ensuring the efficacy of patients, the economic burden of patients is reduced by reducing the cost of materials, shortening the length of hospitalization, and strengthening hospital management, and controlling the continuous growth of hospitalization costs. However, more samples and possible influencing factors could be included for multi-center studies, to provide scientific guidance for improving the rapid growth of hospitalization costs and adjusting the cost structure.

## Author contributions

**Data curation:** Lili Zhao, Jiaji Tang.

**Funding acquisition:** Lili Zhao.

**Investigation:** Lili Zhao.

**Resources:** Lili Zhao.

**Supervision:** Lili Zhao.

**Validation:** Lili Zhao, Jiaji Tang.

**Writing – original draft:** Lili Zhao, Jiaji Tang.

**Writing – review & editing:** Lili Zhao, Jiaji Tang.
